# Clemastine Ameliorates Perioperative Neurocognitive Disorder in Aged Mice Caused by Anesthesia and Surgery

**DOI:** 10.3389/fphar.2021.738590

**Published:** 2021-08-23

**Authors:** Wensi Wu, Xiaojun Zhang, Jiaxin Zhou, Hongmei Yang, Junjun Chen, Le Zhao, Junying Zhong, Wei-jye Lin, Zhi Wang

**Affiliations:** ^1^ Department of Anesthesiology, Sun Yat-Sen Memorial Hospital, Guangzhou, China; ^2^ Guangdong Provincial Key Laboratory of Malignant Tumor Epigenetics and Gene Regulation, Sun Yat-Sen Memorial Hospital, Sun Yat-Sen University, Guangzhou, China; ^3^ Medical Research Center of Sun Yat-Sen Memorial Hospital, Sun Yat-Sen University, Guangzhou, China

**Keywords:** perioperative neurocognitive disorder, clemastine, neuroinflammation, wnt/β-catenin signaling, remyelination, synaptic plasticity

## Abstract

Perioperative neurocognitive disorder (PND) leads to progressive deterioration of cognitive function, especially in aged patients. Demyelination is closely associated with cognitive dysfunction. However, the relationship between PND and demyelination remains unclear. Here we showed that demyelination was related to the pathogenesis of PND. Clemastine, an antihistamine with potency in remyelination, was predicted to have a potential therapeutic effect on PND by next-generation sequencing and bioinformatics in our previous study. In the present study, it was given at 10 mg/kg per day for 2 weeks to evaluate the effects on PND in aged mice. We found that clemastine ameliorated PND and reduced the expression levels of inflammatory factors such as tumor necrosis factor alpha (TNF-α) and interleukin-1 beta (IL-1β). Further investigation suggested clemastine increased the expression of oligodendrocyte transcription factor 2 (OLIG2) and myelin basic protein (MBP) to enhance remyelination by inhibiting the overactivation of the WNT/β-catenin pathway. At the same time, the expression of post-synaptic density protein 95 (PSD95, or DLG4), brain-derived neurotrophic factor (BDNF), synaptosomal-associated protein 25 (SNAP25) and neuronal nuclei (NEUN) were also improved. Our results suggested that clemastine might be a therapy for PND caused by anesthetic and surgical factors in aged patients.

## Introduction

Perioperative neurocognitive disorder (PND) is one of the most common perioperative central nervous system complications in aged patients, which can cause changes in personality, social ability and cognitive function (Evered et al., 2018). Advanced age is considered as an independent risk factor of PND (Luo et al., 2019). The incidence of PND is about 10–54%, which is higher in patients over age of 65. Some of them even developed into dementia in 3–5 years after suffering PND (Punjasawadwong et al., 2018). Poor perioperative cognitive function seriously affects patient’s life quality and increases perioperative complications and mortalities (Jiang et al., 2019). At present, the exact pathogenesis of PND remains unclear. Neuroinflammation is primarily involved in the mechanism and there is a lack of effective treatment.

The maintenance of cognitive function mainly depends on the normal physiological status of neurons. Myelin sheath, the tubular membrane surrounding the axon, playing an important role in maintaining the normal physiological condition of neuron (Del Giovane and Ragnini-Wilson, 2018). First, myelin sheath isolates the axon from the surrounding tissues to avoid interference between nerve impulses (Ravera et al., 2016; Huntemer-Silveira et al., 2020). Second, it increases the conduction speed of nerve impulses (Saab and Nave, 2017). More importantly, myelin sheath has a protective effect on inducing the regeneration of the axon when it comes to injury (Grove et al., 2020). The correct formation of myelin sheath requires the differentiation and maturation of oligodendrocytes (OLs), which is regulated by various signaling pathways, particularly the WNT/β-catenin (Tandon et al., 2020). It suppresses oligodendrogenesis via direct inhibition of OLIG2 expression (Jiang et al., 2020). Meanwhile, neuroinflammation induces axonal hypomyelination through the overactivation of WNT/β-catenin signal pathway (Huang et al., 2020). In other words, neuroinflammation is one of the main factors leading to demyelination (Huang et al., 2020; das Neves et al., 2020; Borgonetti et al., 2020). Demyelination pathological changes widely exist in many kinds of neurodegenerative, brain injury and cognitive impairment diseases, such as Alzheimer’s disease, multiple sclerosis, stroke, traumatic brain injury and spinal cord injury (Mayne et al., 2020). It is worth noting that no studies have been conducted on the relationship between PND and demyelination. On the contrary, remyelination contributes to the improvement of cognitive function (Del Giovane and Ragnini-Wilson, 2018; Chen et al., 2021a), which might be helpful in the treatment of PND. Besides, neuronal function can also benefit from remyelination and mainly manifested by synaptic function (Del Giovane and Ragnini-Wilson, 2018). Synaptic plasticity is one of the criterions for evaluating synaptic function. Previous studies have confirmed that PND is closely related to the impairment of synaptic plasticity (Gao et al., 2021).

The antihistamine clemastine, an FDA-approved drug with high potency in enhancing remyelination, anti-neuroinflammatory and brain function improvement in a variety of diseases including hypoxic-ischemic encephalopathy, aging, multiple sclerosis and depression (Liu et al., 2016; Green et al., 2017; Cree et al., 2018; Su et al., 2018; Wang et al., 2020a; Xie et al., 2020). Meanwhile, clemastine ameliorates cognitive impairment in mice caused by early postnatal exposure to isoflurane through enhancing remyelination (Li et al., 2019). However, the effect of clemastine on PND is unknown. In our previous study, clematine was predicted to have a potential therapeutic effect on PND through next-generation sequencing combined with bioinformatics analysis (Wu et al., 2021).

Thus, we hypothesized that clemastine ameliorated the impairment of learning and memory induced by anesthesia and surgery in aged mice. To test these hypotheses, we performed surgery and anesthesia on aged (18-month-old) male C57BL/6 mice and explored the effect of clemastine on PND.

## Materials and Methods

### Animals

**FIGURE 1:**
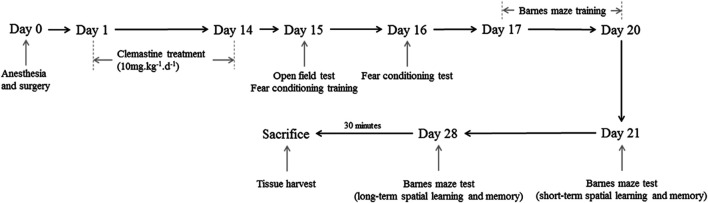
Diagram of timeline of experimental procedures.

Male C57BL/6 mice at 18 months and weighing 45–50 g were supplied by Sun Yat-sen University (Guangzhou, China). These mice were housed in specific pathogen free environment kept at 19–23°C and 40–60% humidity with a 12-h light/12-h dark cycle (light from 07:00 to 19:00). The mice were grouped into four categories in a random manner: control (CON), control plus clemastine group (CON + CLE), PND alone (PND), and PND plus clemastine group (PND + CLE) (*n* = 20, each group). Five animals were kept in each cage and allowed to have food and water ad libitum. The experiment started until all animals had adapted to the environment for 2 weeks. All the animal experiments in the present study were approved by the Institutional Animal Care and Use Committee (Approval No: SYSU-IACUC-2020-000326) and the Laboratory Animal Ethics Committee of Sun Yat-sen University. All procedures were performed in accordance with the approved guidelines. The schematic timeline of the experimental process is shown in Figure 1.

### Animal Model

Isoflurane anesthesia plus exploratory laparotomy has been proved to be an effective method to construct PND model (Qiu et al., 2020). Before exploratory laparotomy, the mice were anesthetized by exposing to an oxygen chamber prefilled with 1.5% isoflurane for 30 min. A median incision approximately 2  cm in the abdomen was made to enter the abdominal cavity and explore the abdominal organs such as the liver, spleen, and intestine. Sterile 5–0 surgery sutures were used to suture the peritoneum and skin. Surgery was also performed with isoflurane inhalation anesthesia and lasted 30 min. As shown in our previous study, mice received this surgery with isoflurane inhalation anesthesia had no hypoxia, heart rates and respiratory rates were kept within the normal ranges (Wu et al., 2021). During the whole process, an anesthesia monitor (B450, GE, United States) was used to dynamically monitor the depth of anesthesia and maintain the anesthesia level when toe pinch and surgery did not respond. Meanwhile, the rectal temperature was monitored and maintained at 37°C with the aid of a heating blanket (69,020, RWD, CHN). At the end of surgery and every day within 3 days after surgery, 2.5% lidocaine cream was applied to the incision to alleviate the postoperative pain, and povidone iodine solution was applied to prevent infection. For the mice that served as controls, neither anesthesia nor surgery was performed.

### Drug

From the first day after anesthesia and surgery, aged mice in CON + CLE group and PND + CLE group were intraperitoneally injected with clemastine (C129211, Aladdin, CHN) at 10 mg/kg per day for 2 weeks. Clemastine was prepared freshly each day by dissolving the powder in normal saline. The selected dose is based on previous studies, which proves that clemastine has the effect of enhancing cognition and remyelination (Li et al., 2019; Chen et al., 2021b; Li et al., 2021). Aged mice in the CON and PND group received an intraperitoneal injection of the same amount of normal saline every day to ensure the consistency of the experiment.

### Behavioral Studies

All behavior tests were performed in a sound-isolated room between 12:00 and 18:00. All behavioral data was recorded by the same two researchers who were blinded to the animal grouping.

### Open Field

A black opaque plastic chamber (60 × 60 × 50 cm, ZH-ZFT, Anhui Zhenghua Biological Instrument equipments Co., Ltd., Anhui, China) was used as the open field arena. On the first day after 2 weeks of clemastine treatment, the open field test was performed to evaluate the locomotor activity and postoperative anxiety of the mice (Qiu et al., 2020). Each mouse was placed in the center of the field and allowed to explore freely for 5 min with a video tracking system (Smart v3.0.06, Panlab Harvard Apparatus, Barcelona, ES) automatically recorded its movements, analyzed the total distance in the whole area and the time spent in the center area. During each test interval, the field was cleaned with 75% ethanol to eliminate feces and odors.

### Fear Conditioning

Two hours after the open field test, each mouse was placed into the conditioning chamber (Freeze Monitor, San Diego Instruments, San Diego, CA) and allowed to explore the room freely for 180 s. Then they were given a 30 s tone (70 db), followed by a 2 s foot shock (0.7 mA), and the next tone-shock stimulation cycle was entered at an interval of 60 s. A total of 3 cycles were performed as training. One day later, each mouse was placed into the conditioning chamber without any tone or electrical stimulation for 360 s and the environment was identical with that before, the time of freezing behavior was recorded to test the context-related memory (hippocampus-dependent memory). 2 hours later, they were placed into a new environment completely different from that before and explored room freely for 180 s. Then they were given the same tone stimulation as before (without electrical stimulation), the time of freezing behavior was recorded to test the tone-related memory (hippocampus-independent memory). Freezing behavior means there is no visible movement other than breathing (Qiu et al., 2016). During each test interval, the conditioning chamber was cleaned with 75% ethanol to eliminate feces and odors.

### Barnes Maze

2 hours after the fear conditioning test, each mouse was placed in a small dark recessed chamber to acclimate for 5 minutes. Over the next 12 days, we performed Barnes maze to evaluate the spatial learning and memory of mice (Zheng et al., 2017). In the first 4 days, each mouse was placed in the center of a circular platform (Anhui Zhenghua Biological Instrument equipments Co., Ltd., Anhui, China) with a diameter of 92 cm, which had 20 equally spaced holes. Among all the holes, only one was linked to the dark chamber. The mice were expected to find the hole and enter the dark chamber under the bright light (200 W). The mice were trained for 4 days with 3 trials per day, with each trial lasting 3 min, and an interval of 15 min between each trial. If the mice could not find the correct hole and enter the dark chamber over 3 min, they would be guided to the correct location. During each test interval, the platform and dark chamber were cleaned with 75% ethanol to eliminate feces and odors. On the 5th day, the escape latency and the number of wrong holes explored were recorded and measured by a video tracking system (Smart v3.0.06, Panlab Harvard Apparatus, Barcelona, ES), which was used to evaluate the short-term spatial learning and memory of mice. The escape latency is the time a mouse used to enter the dark chamber. A week later, the mice were tested for long-term spatial learning and memory in the same way.

### Sequencing Data and Identification of Differentially Expressed Genes

The gene expression profile data (accession number GSE174413) which we previously obtained through next-generation sequencing was downloaded from the Gene Expression Omnibus (GEO) database. It contains the gene expression profiles of the brain tissues of three aged male C57BL/6 mice in the PND group and the CON group, respectively (Wu et al., 2021). Limma package in R software was applied to screen differentially expressed genes of PND with *p*-value < 0.05, | log2 (Fold Change) | ≥ 1. By processing the ggpubr and ggthemes package of R software, we accomplished the visualization of differentially expressed genes.

### Identification of Protein-Protein Interactions

The Search Tool for the Retrieval of Interacting Genes (STRING, version 11.0, https://www.string-db.org/) database was used to identify protein-protein interactions between genes. The protein-protein minimum required interaction score was set to 0.4, indicating medium confidence.

### Harvesting of Brain Tissue

Mice were deeply anesthetized with isoflurane 30 min after the Barnes maze test (long-term spatial learning and memory test), and perfused transcardially with normal saline (*n* = 10, each group). The brain was dissected in 4°C environment and stored at 80°C before use. The hippocampus was isolated for subsequent experiments on genes and proteins levels. Other mice were perfused transcardially with normal saline and 4% paraformaldehyde, and further fixed with 4% paraformaldehyde for 24 h (*n* = 10, each group). Then dehydrated with a gradient of 10, 20, and 30% sucrose for 1 day each until the brain was completely sunk to the bottom. Absorbed the moisture on the surface and used optimum cutting temperature (OCT) compound (4583, SAKURA, JP) for embedding in subsequent immunofluorescence staining.

### Reverse Transcription-Quantitative Polymerase Chain Reaction

Hippocampus was lysed and total RNA was extracted using RNA Quick Purification kit (RN001, ESscience, CHN). The concentrations of the RNA samples were determined spectrophotometrically at 260, 280, and 230 nm by using a NanoDrop ND-2000 (Thermo, United States) instrument. Total RNA was subsequently reverse transcribed into cDNA using Hifair^®^ III 1st Strand cDNA Synthesis SuperMix for qPCR (11141ES60, Yeasen, CHN). The reverse transcription conditions were as follows: 25°C for 5 min, 55°C for 15 min and 85°C for 5 min. The qPCR was performed using Hieff UNICON^®^ qPCR SYBR^®^ Green Master Mix (11198ES08, Yeasen, CHN) on Roche LightCycler 480 II Real-Time PCR System (Roche, United States). The thermocycling conditions were as follows: Initial denaturation at 95°C for 30 s, followed by 40 cycles at 95°C for 10 s, 60°C for 20 s and 72°C for 20 s. The data were analyzed using the 2−ΔΔCt method. GAPDH was used as an internal control. The sequences of primers are presented in Supplementary Table S1.

### Western Blot Analysis

Hippocampus was lysed with RIPA buffer (P0013B, Beyotime, CHN) and protein concentration was determined using a BCA protein quantification kit (P0010, Beyotime, CHN). Proteins were separated by SDS-PAGE (P0012A, Beyotime, CHN) using 10% gels and transferred to PVDF membranes (ISEQ00010, Merck Millipore, United States). After blocking with 5% skimmed milk (A600669, Sangon Biotech, CHN) for 1 h at room temperature, PVDF membranes were incubated with primary antibodies at 4°C overnight, followed by incubation with secondary antibodies at room temperature for 1 h. Subsequently, the protein bands were visualized using ECL reagent (WBKLS0100, Merck Millipore, United States), and quantitated with ImageJ software (National Institutes of Health, Bethesda, MD, United States). Rabbit polyclonal anti-mouse TNF-α antibody (1:1,000, AF8208, Beyotime, CHN), rabbit polyclonal anti-mouse IL-1β antibody (1:1,000, AF7209, Beyotime, CHN), rabbit polyclonal anti-mouse WNT10B antibody (1:1,000, DF9038, Affinity, CHN), rabbit polyclonal anti-mouse β-catenin antibody (1:1,000, AF5126, Beyotime, CHN), rabbit monoclonal anti-mouse OLIG2 antibody (1:1,000, AF1312, Beyotime, CHN), rabbit polyclonal anti-mouse MBP antibody (1:1,000, BA0094, Boster, CHN), rabbit polyclonal anti-mouse SNAP25 antibody (1:1,000, AF8016, Beyotime, CHN), rabbit monoclonal anti-mouse PSD95 antibody (1:1,000, AF1096, Beyotime, CHN), rabbit monoclonal anti-mouse BDNF antibody (1:1,000, AF1423, Beyotime, CHN) and rabbit monoclonal anti-mouse β-tubulin antibody (1:1,500, AF1216, Beyotime, CHN) were the primary antibodies used. The secondary antibodies used were horseradish peroxidase (HRP) - conjugated goat anti-rabbit lgG (1:1,500, A0208, Beyotime, CHN).

### Immunofluorescence Assay

Brain tissues were embedded with OCT and sliced into 10 μm. The sections were washed three times with phosphate buffer solution (PBS) to remove OCT from the surface. After blocking with goat serum (16210072, Gibco, United States) for 1 h at room temperature, tissue sections were incubated with mouse monoclonal anti-mouse NEUN antibody (1:100, MAB377, Merck millipore, GER) and rabbit polyclonal anti-mouse MBP antibody (1:100, BA0094, Boster, CHN) at 4°C overnight. After rewarming for 1 h, tissue sections were washed three times with PBS, incubated with donkey anti-mouse IgG (H + L) highly cross-adsorbed secondary antibody, alexa fluor 647 (1:1,000, A-31571, Invitrogen, United States) and CY3-labeled goat anti-mouse IgG (1:500, A0562, Beyotime, CHN) at room temperature for 2 h, and incubated with DAPI (G1012, Servicebio, CHN) at room temperature for 10 min. Tissue sections were sealed with anti-fluorescence quenching reagent (P0128M, Beyotime, CHN) and images were obtained using a laser confocal microscope (×200, magnification) (Zeiss LSM 800 with airyscan, GER).

### Statistical Analysis

All data were expressed as mean ± S.D. The statistical analysis of results was performed by using GraphPad Prism version 8.0 (San Diego, CA, United States) and R software. The inter-group comparisons were assessed by one-way repeated measures analysis of variance followed by Tukey test. The data of training sessions in the Barnes maze test was analyzed by two-way repeated measures analysis of variance followed by Tukey test. A *p*-value < 0.05 was considered statistically significant difference.

## Results

### Clemastine Ameliorated Perioperative Neurocognitive Disorder Caused by Anesthesia and Surgery in Aged Mice

**FIGURE 2:**
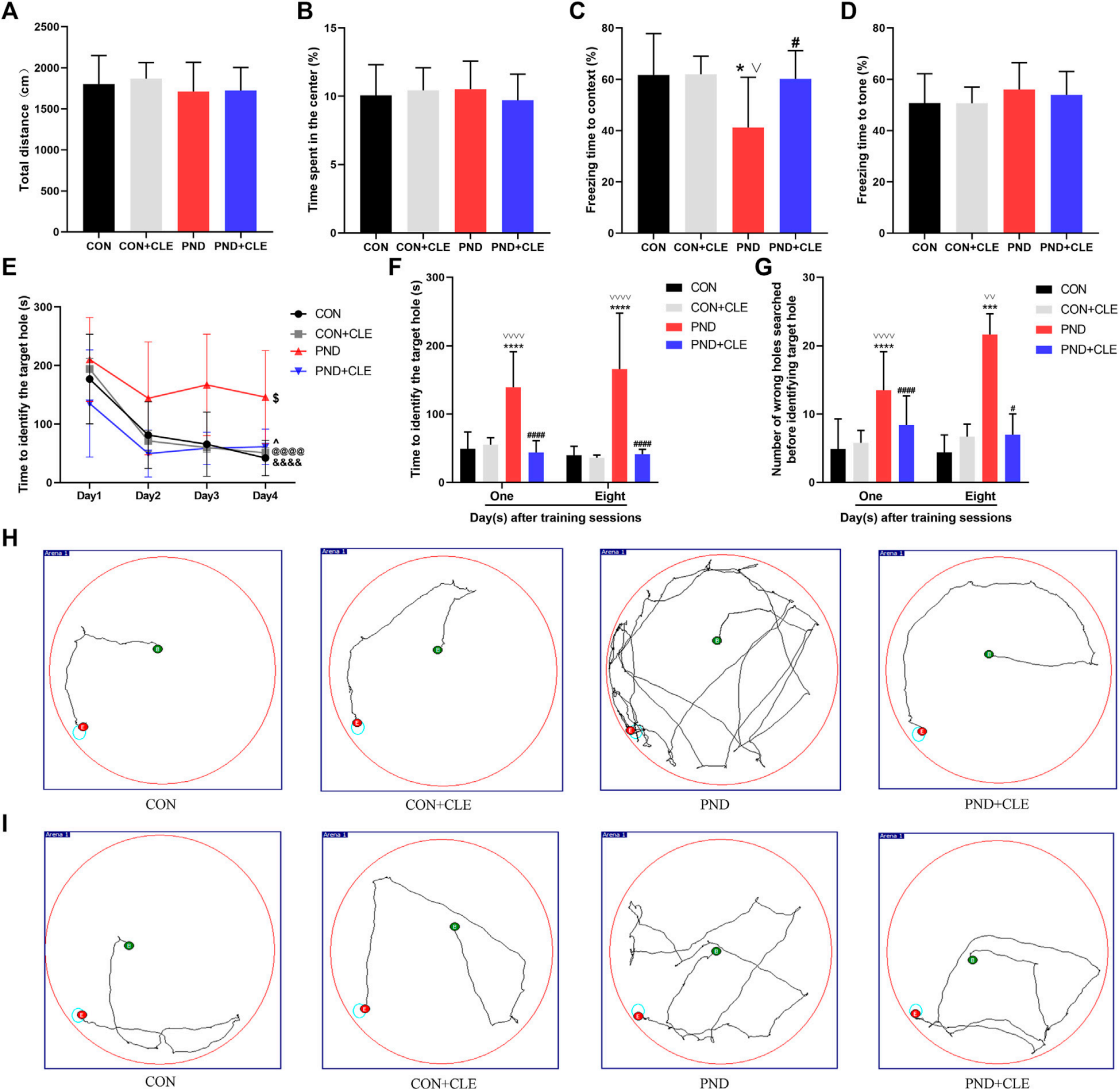
Anesthesia and surgery-induced cognitive impairments were ameliorated by clemastine treatment. (**A**) Total distance in the open field test among four groups. (**B**) Time spent in the center of open field test among four groups. (**C**) Context test in the fear conditioning test among four groups. (**D**) Tone test in the fear conditioning test among four groups. (**E**) Performance during the training phase of the Barnes maze. (**F, G**) Performance during the testing phase of Barnes maze. (**H, I**) Representative movement traces on day 1 and day 8 of the Barnes maze test phase. The data are presented as mean ± S.D. (*n* = 20 mice per group). ^*^ *p* < 0.05 compared with the CON group. ***p < 0.005 compared with the CON group. ****p < 0.001 compared with the CON group. ^#^ *p* < 0.05 compared with the PND group. ^####^ *p* < 0.001 compared with the PND group. ^∨^ *p* < 0.05 compared with the CON + CLE group. ^∨∨^ *p* < 0.01 compared with the CON + CLE group. ^∨∨∨∨^ *p* < 0.001 compared with the CON + CLE group. ^&&&&^ *p* < 0.001 compared with the day 1 in CON group. ^@@@@^ *p* < 0.001 compared with the day 1 in CON + CLE group. ^$^ *p* < 0.05 compared with the first day in PND group. ^^^ *p* < 0.05 compared with the first day in PND + CLE group.

In the behavioral test, we first evaluated the locomotor activity and postoperative anxiety behavior of aged mice in different groups through the open field test. The results showed that anesthesia plus surgery or clemastine had no influence on the locomotor activity of the aged mice, nor did it cause the occurrence of postoperative anxiety behavior (Figures 2A,B). Second, we assessed the changes in hippocampal and non-hippocampal memory in aged mice through fear conditioning test. The results showed that anesthesia and surgery-induced hippocampus-dependent memory defects were improved after treatment with clemastine (Figures 2C,D). Finally, we estimated the effects of anesthetic and surgical factors on short-term and long-term spatial learning and memory ability of aged mice through Barnes maze test. The time required for all the aged mice to find the target hole on the 4th day of training phase were significantly reduced compared to the first day, indicating that all mice achieved performance development after training (Figure 2E). On the first and eighth days of the test phase, we found that the PND group took more time to find the target hole and explored more error holes than the CON group and CON + CLE group. However, the PND + CLE group used shorter time and explored less error holes compared with PND group (Figures 2F–I). These results suggested that clemastine could effectively ameliorate PND caused by anesthesia and surgery in aged mice. It is noteworthy that in the above-mentioned tests, clemastine treatment showed no effect on the aged mice of CON group, which was also confirmed in the previous study (Li et al., 2021). In the follow-up studies, brain tissues of aged mice in CON group, PND group and PND + CLE group were harvested to explore the possible mechanism.

### Clemastine Exhibited Anti-neuroinflammatory Effects in Aged Mice With Perioperative Neurocognitive Disorder

**FIGURE 3:**
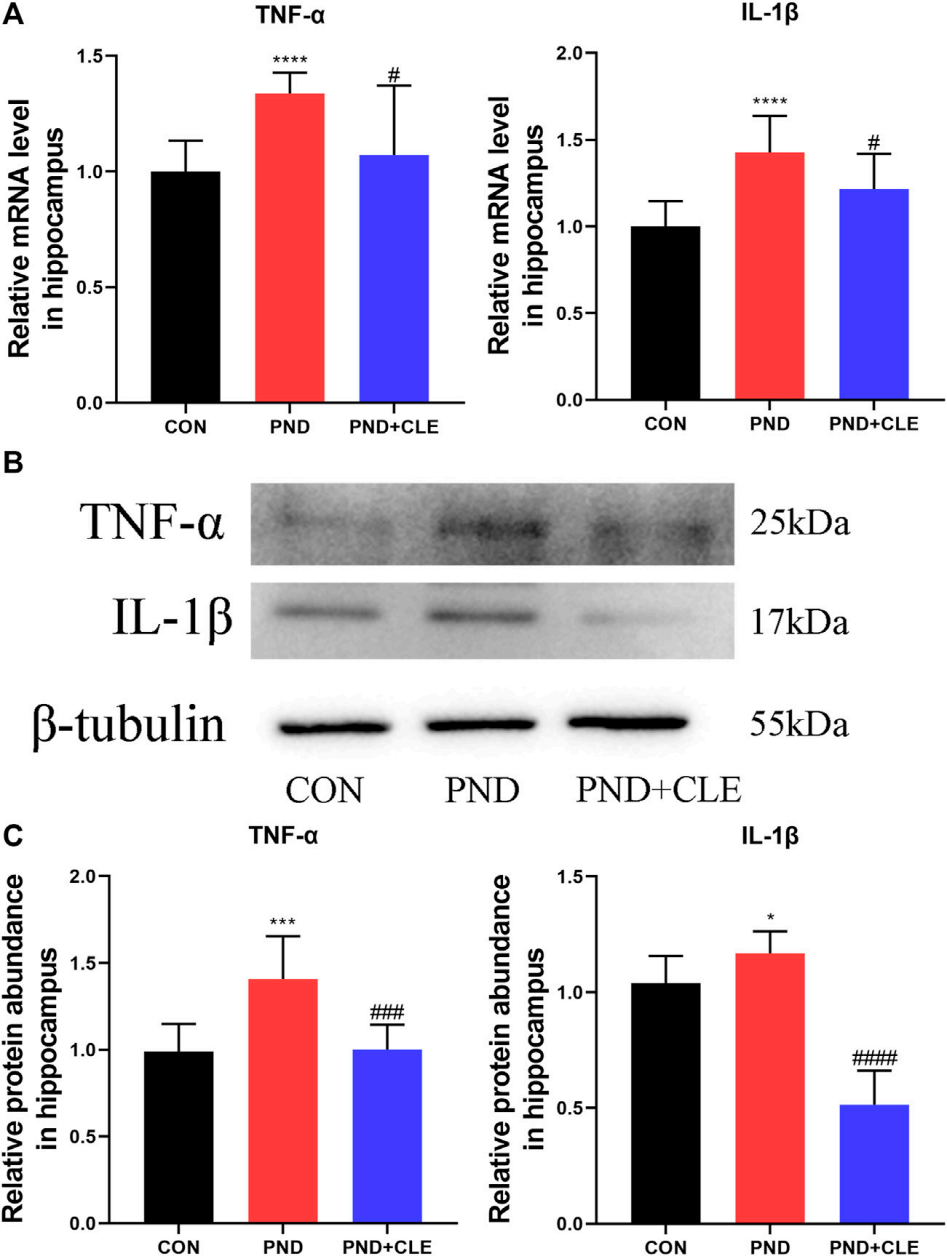
Effects of clemastine on expression levels of inflammatory cytokines in hippocampus of aged mice after anesthesia and surgery. (**A**) Relative mRNA expressions of TNF-α and IL-1β, normalized to that of the GAPDH internal control. (**B**) Representative western blot images of TNF-α and IL-1β. (**C**) Relative protein expressions of TNF-α and IL-1β, normalized to that of the β-tubulin internal control. The data are presented as mean ± S.D. (*n* = 10 mice per group). ^*^ *p* < 0.05 compared with the CON group. ***p < 0.005 compared with the CON group. ****p < 0.001 compared with the CON group. ^#^ *p* < 0.05 compared with the PND group. ^###^ *p* < 0.005 compared with the PND group. ^####^ *p* < 0.001 compared with the PND group.

Neuroinflammation is primarily involved in the pathological mechanism of PND (Liu et al., 2021). In the present study, we measured the expression levels of TNF-α and IL-1β in the hippocampus of aged mice. The results showed the expression of TNF-α and IL-1β were up-regulated in the PND group compared with the CON group. Besides, the expressions were down-regulated in the PND + CLE group compared with the PND group (Figures 3A–C). The results showed that clemastine, as a histamine H1 receptor antagonist, effectively reduced neuroinflammatory in aged mice with PND.

### Clemastine Inhibited the Overactivation of WNT/β-Catenin Pathway in Aged Mice With Perioperative Neurocognitive Disorder

**FIGURE 4:**
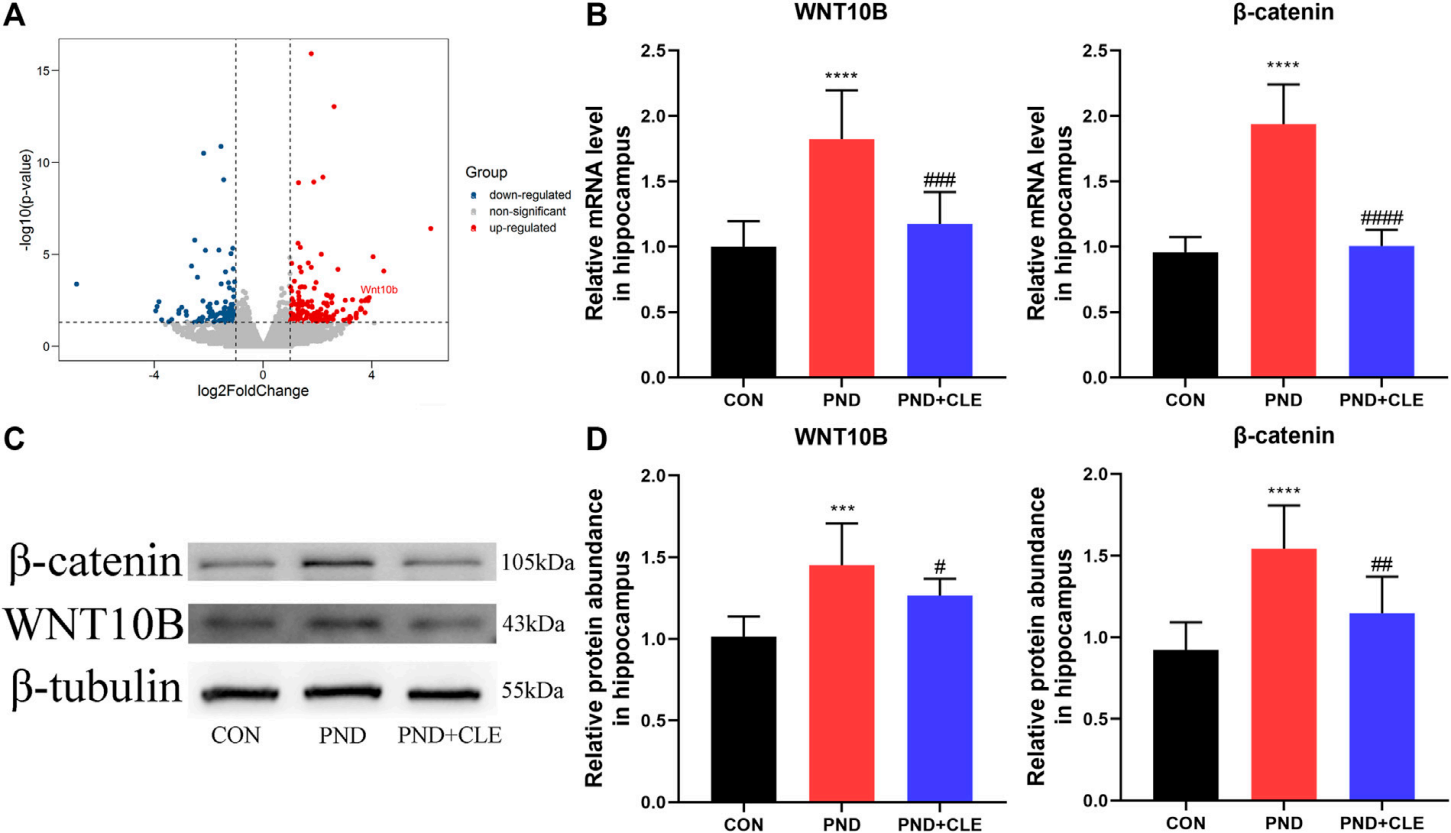
Effects of clemastine on expression levels of WNT/β-catenin signaling pathway. (**A**) The differential expression of WNT10B was labeled in our previous sequencing results. (**B**) Relative mRNA expressions of WNT10B and β-catenin, normalized to that of the GAPDH internal control. (**C**) Representative western blot images of WNT10B and β-catenin. (**D**) Relative protein expressions of WNT10B and β-catenin, normalized to that of the β-tubulin internal control. The data are presented as mean ± S.D. (*n* = 10 mice per group). ***p < 0.005 compared with the CON group. ****p < 0.001 compared with the CON group. ^#^ *p* < 0.05 compared with the PND group. ^##^ *p* < 0.01 compared with the PND group. ^###^ *p* < 0.005 compared with the PND group. ^####^ *p* < 0.001 compared with the PND group.

Neuroinflammation leads to overactivation of the WNT/β-catenin signaling pathway (Huang et al., 2020; Vallée et al., 2018). In our previous sequencing results, the expression of WNT10B (member of the WNT ligand gene family) in aged PND mice was significantly up-regulated (Figure 4A) (Wu et al., 2021). It indicated the overactivation of WNT/β-catenin signaling pathway. In the present study, we measured the expression levels of WNT10B and β-catenin in the hippocampus of aged mice. The results showed the expression of WNT10B and β-catenin were up-regulated in the PND group compared with the CON group. Besides, the expressions were down-regulated in the PND + CLE group compared with the PND group (Figures 4B–D). The results showed that clemastine inhibited the overactivation of WNT/β-catenin signaling pathway in aged mice with PND.

### Clemastine Enhanced OLs Differentiation and Remyelination in Aged Mice With Perioperative Neurocognitive Disorder

**FIGURE 5:**
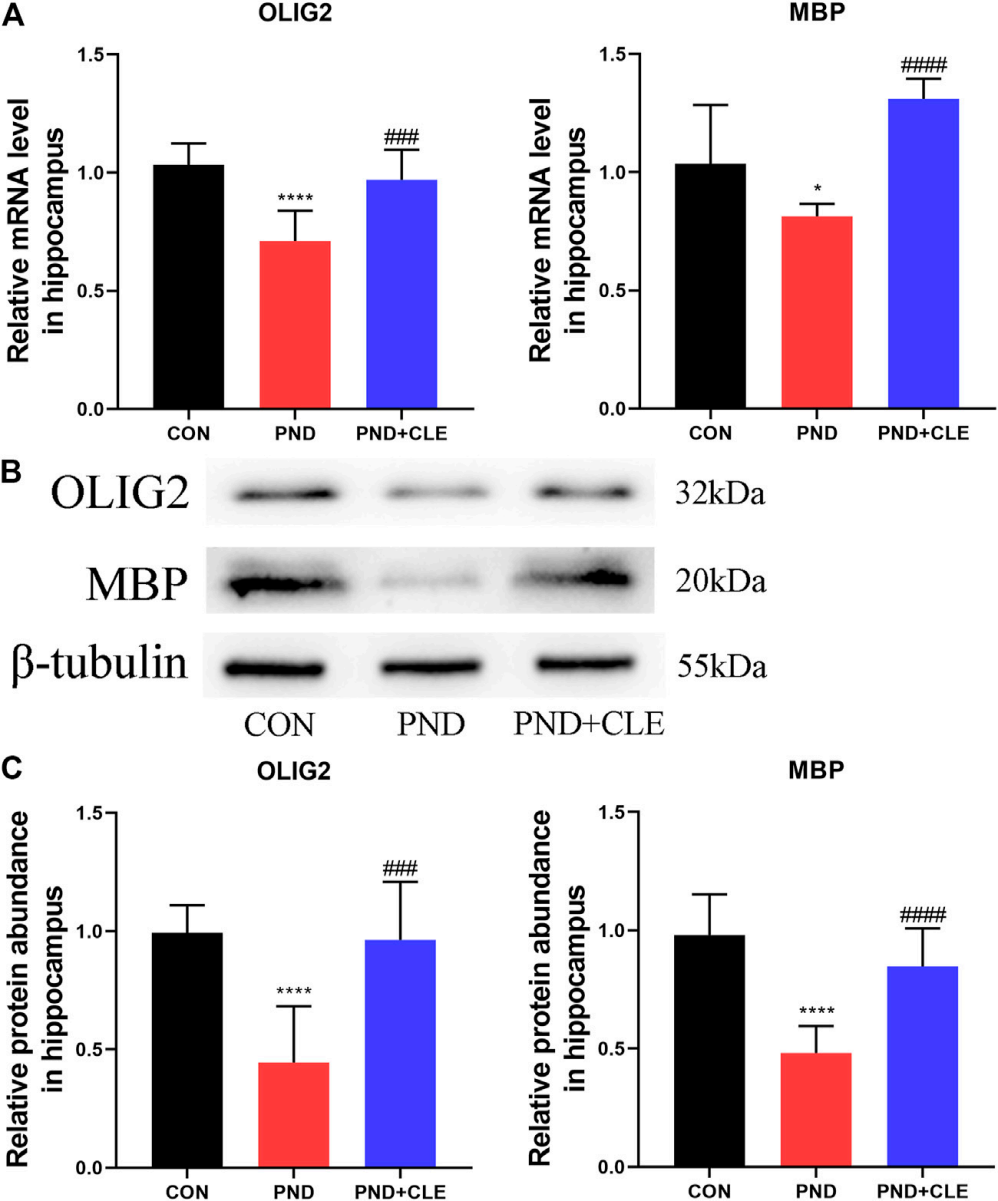
Effects of clemastine on expression levels of OLIG2 and MBP in hippocampus of aged mice after anesthesia and surgery. (**A**) Relative mRNA expressions of OLIG2 and MBP, normalized to that of the GAPDH internal control. (**B**) Representative western blot images of OLIG2 and MBP. (**C**) Relative protein expressions of OLIG2 and MBP, normalized to that of the β-tubulin internal control. The data are presented as mean ± S.D. (*n* = 10 mice per group). ^*^ *p* < 0.05 compared with the CON group. ****p < 0.001 compared with the CON group. ^###^ *p* < 0.005 compared with the PND group. ^####^ *p* < 0.001 compared with the PND group.

The overactivation of WNT/β-catenin signaling pathway is detrimental to the OLs differentiation and remyelination (Huang et al., 2020; Jiang et al., 2020). The expression of OLIG2 and MBP represent the levels of OLs differentiation and remyelination, respectively. We found that the expression of OLIG2 and MBP were down-regulated in the PND group compared with the CON group, while upregulated in PND + CLE group (Figures 5A–C). These results showed that clemastine effectively enhanced OLs differentiation and remyelination in aged mice with PND.

### Clemastine Reversed the Dysregulation of Synaptic Plasticity-Related Proteins in Aged Mice With Perioperative Neurocognitive Disorder

**FIGURE 6:**
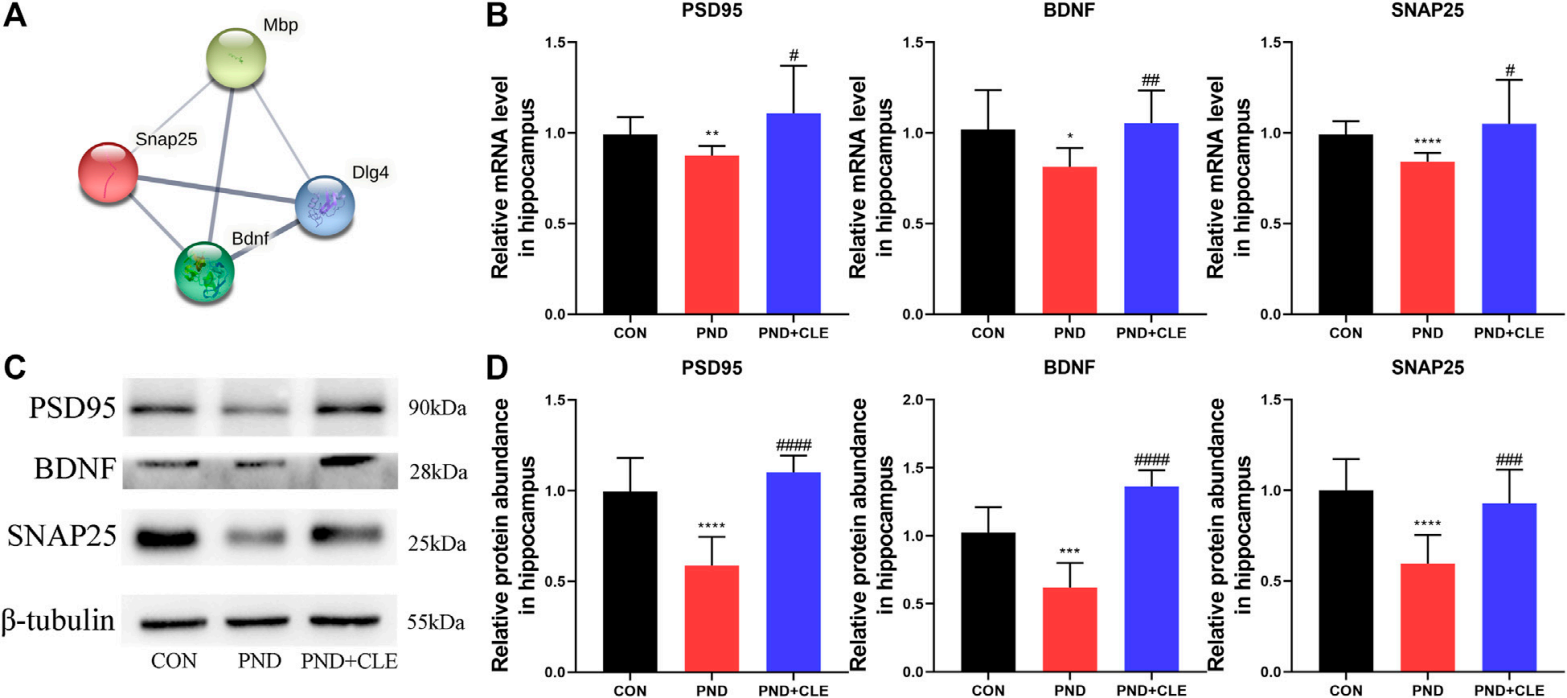
Effects of clemastine on expression levels of synaptic plasticity-related proteins in hippocampus of aged mice after anesthesia and surgery. (**A**) Co-expression relationship of MBP, PSD95 (or DLG4), BDNF and SNAP25 from the STRING database. (**B**) Relative mRNA expressions of PSD95, BDNF and SNAP25, normalized to that of the GAPDH internal control. (**C**) Representative western blot images of synaptic plasticity-related proteins. (**D**) Relative protein expressions of PSD95, BDNF and SNAP25, normalized to that of the β-tubulin internal control. The data are presented as mean ± S.D. (*n* = 10 mice per group). ^*^ *p* < 0.05 compared with the CON group. **p < 0.01 compared with the CON group. ***p < 0.005 compared with the CON group. ****p < 0.001 compared with the CON group. ^#^ *p* < 0.05 compared with the PND group. ^##^ *p* < 0.01 compared with the PND group. ^###^ *p* < 0.005 compared with the PND group. ^####^ *p* < 0.001 compared with the PND group.

Remyelination helps restore the neuronal function and prevents neurodegeneration (Del Giovane and Ragnini-Wilson, 2018). PSD95, BDNF and SNAP25 are the synaptic plasticity-related proteins that closely related to neuronal function, learning and memory (Qiu et al., 2020; Jia et al., 2021; Muscat et al., 2021). Protein-protein interaction analysis results indicated that there were co-expression relationships between MBP, PSD95, BDNF and SNAP25 (Figure 6A). The results showed, anesthesia and surgery decreased the expression levels of synaptic plasticity-related proteins, clemastine could reverse the down-regulation (Figures 6B–D). These results showed that clemastine could improve synaptic plasticity in aged mice with PND.

### Clemastine Prevented the Loss of Hippocampal Mature Neurons in Aged Mice With Perioperative Neurocognitive Disorder

**FIGURE 7:**
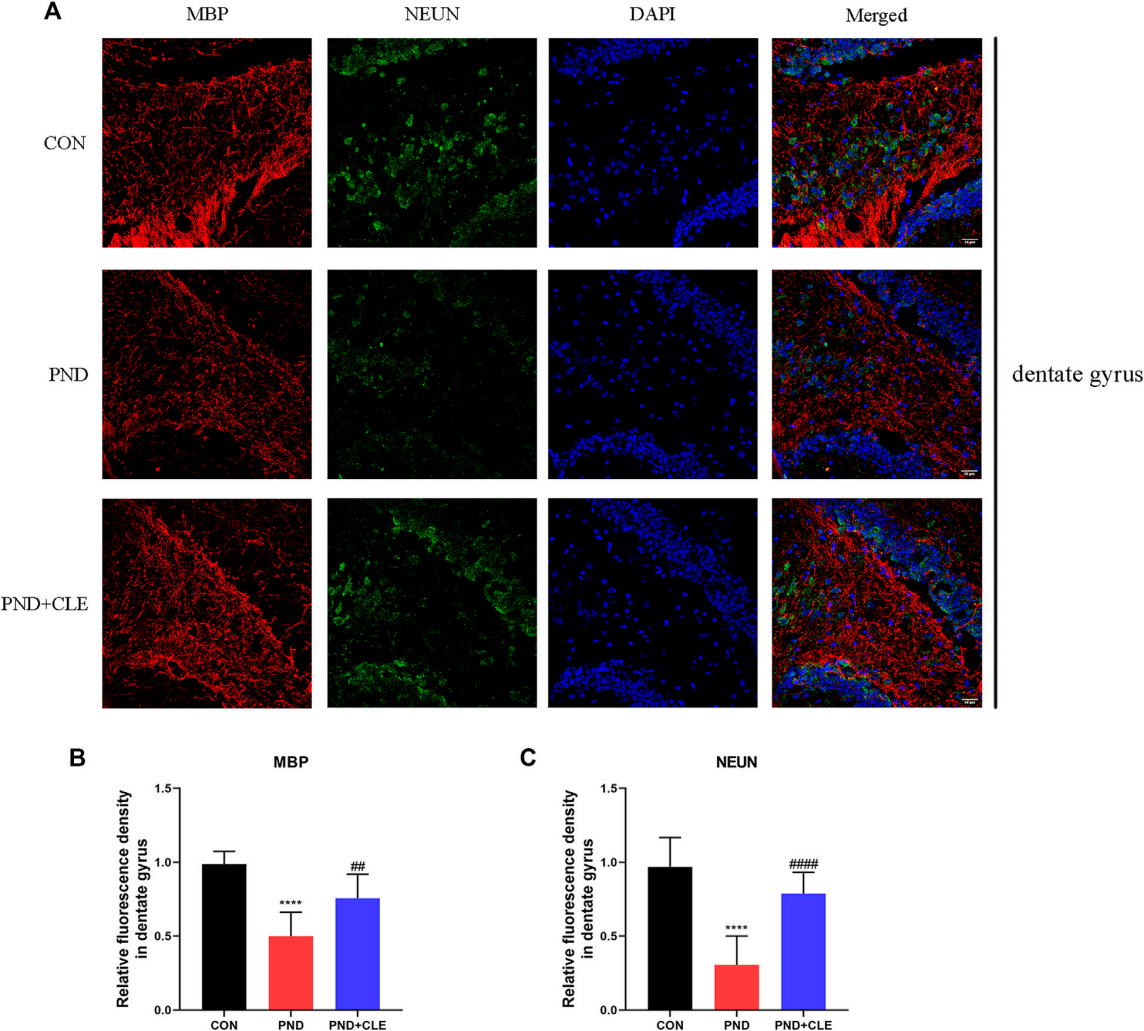
Immunofluorescence analysis detected MBP and NEUN protein levels in hippocampal dentate gyrus (scale bar = 50 µm). (**A**) Representative images of MBP and NEUN in the dentate gyrus. (**B**) Mean fluorescence density of MBP in the dentate gyrus. (**C**) Mean fluorescence density of NEUN in the dentate gyrus. The data are presented as mean ± S.D. (*n* = 10 mice per group). ****p < 0.001 compared with the CON group. ^##^ *p* < 0.01 compared with the PND group. ^####^ *p* < 0.001 compared with the PND group.

Synaptic plasticity is related to the neuronal survival (Labandeira et al., 2021; Limanaqi et al., 2021). Neuronal loss is also one of the main pathological mechanisms of PND (Zhang et al., 2016). In the present study, similar to the expression level of MBP, immunofluorescence results found that NUEN in the hippocampal dentate gyrus of PND group was significantly decreased compared with the CON group, while clemastine reversed the decrease (Figures 7A–C). These results showed that clemastine prevented the loss of hippocampal mature neurons in aged mice with PND.

## Discussion

PND is a complication of the central nervous system in elderly patients after surgery, which manifests as psychosis, anxiety, personality changes and impaired memory (Evered et al., 2018). Cardiac surgery and some non-cardiac surgery, such as abdominal and chest surgery, are associated with a high incidence of PND (Newman et al., 2001). In addition, age is an independent risk factor in the occurrence and development of PND (Moller et al., 1998). In elderly patients over 60 years of age, the incidence of PND is 10–62%, which increases the risk of Alzheimer’s disease and may related to dementia (Evered et al., 2016; Urits et al., 2019). In this study, we performed exploratory laparotomy plus isoflurane inhalation anesthesia in 18-month-old male C57BL/6 mice to establish the PND model. Behavioral tests suggested that anesthetic and surgical factors impaired the spatial learning memory in aged mice, including short-term and long-term memory related to the hippocampus. But locomotor activity and postoperative anxiety behavior were not affected. In our previous study, the differentially expressed genes in the brain tissues of aged PND mice were identified by next-generation sequencing, and clematine was predicted to have the potential to treat this refractory disease (Wu et al., 2021). In the present study, we found that clemastine treatment ameliorated the impaired hippocampal-related memories caused by surgical and anesthetic factors. At the same time, it did not cause behavioral changes in normal aged mice, which has been confirmed in the previous study (Li et al., 2021). The results indicated that clemastine had a positive effect on PND in aged mice.

Various mechanisms are involved in the pathogenesis of PND, such as neuroinflammation, oxidative stress and neurodegeneration. The pathophysiological changes caused by neuroinflammation of the central nervous system are the main mechanism of PND (Granger and Barnett, 2021; Liu et al., 2021). In the present study, we found that the expression of TNF-α and IL-1β in the hippocampus increased significantly in the PND group compared with the CON group. It was consistent with previous studies, which have confirmed the association between PND and neuroinflammation (Qiu et al., 2020). Anti-neuroinflammatory therapy has shown some positive effects on the treatment of PND. Previous studies have shown that inhibiting neuroinflammatory by reducing the expression of interleukin-6, C-reactive protein and matrix metalloproteinase-9 in serum can significantly alleviate PND in aged patients (Zhang et al., 2018). The activation of microglia is the key factor in aggravating neuroinflammation, especially the M1 type (Lee et al., 2017). Lipopolysaccharide and sevoflurane treatment induced up-regulation of IL-1β and IL-6 expression in microglia *in vitro* (Ye et al., 2013). *In vivo* experiment, isoflurane induced microglial inflammation and cognitive impairment in aged mice through the NLRP3-Caspase-1 pathway (Wang et al., 2018). At the same time, inhibition of NF-kB/MAPKs pathway of microglia by upregulating the expression of interleukin-10 to improve PND through anti-inflammatory effect (Zhang et al., 2019). Clemastine, as an antihistamine, has a positive anti-neuroinflammatory effect, could reduce the activation of microglia and down-regulate the expression of IL-1β and NLRP3 in rats with hypoxic-ischemic brain injury (Xie et al., 2020). Besides, it also reduced the expression of TNF-α and IL-1β in the hippocampus and serum of depression mice, and inhibited the M1-like activation of microglia (Su et al., 2018). In the present study, we found that clemastine reduced the expression of TNF-α and IL-1β in the hippocampus of aged mice with PND, suggesting the inhibition of neuroinflammation, which is contributed to the treatment of PND.

Neuroinflammation could lead to overactivation of WNT/β-catenin signaling pathway (Xie et al., 2016; Huang et al., 2020). Previous studies have shown that dysregulation of the WNT/β-catenin pathway is closely related to PND (Hu et al., 2016). Our previous sequencing results showed the expression of WNT10B were significantly up-regulated in aged mice with PND (Wu et al., 2021), indicating that PND might induce the overactivation of the WNT/β-catenin pathway. Besides, previous studies demonstrated that the overactivation of WNT/β-catenin pathway leads to the decrease of OLIG2 (Huang et al., 2020; Jiang et al., 2020). The normal expression of OLIG2 contributes to the developmental regulation of oligodendrocytes precursor cells and the early directed differentiation to OLs. OLs are widely distributed in the central nervous system, which contribute to the formation of myelin sheath and could be impaired by neuroinflammation (Xie et al., 2016; Huntemer-Silveira et al., 2020; Joerger-Messerli et al., 2021). In this study, we confirmed PND increased the expression of WNT10B and β-catenin and decreased the expression of OLIG2. Clemastine could reverse the above phenomenon, which contributes to the remyelination (Vallée et al., 2018).

Myelin sheath is formed by OLs and wraps around the outside of axons, accelerating nerve excitatory conduction along nerve fibers and ensuring directional conduction, which is an indispensable process in the development and normal function of nervous system neurons (Ravera et al., 2016; Saab and Nave, 2017; Grove et al., 2020; Huntemer-Silveira et al., 2020). At the same time, myelin sheath regulates the ionic environment and promotes neuron survival through meeting neuronal energy requirements by its metabolites (El Waly et al., 2014; Saab and Nave, 2017). Neuroinflammation is one of the main causes of demyelination. Multiple sclerosis, a chronic inflammatory disease of the central nervous system, characterized by demyelination, can lead to neurodegeneration and neurological function’s impairment (das Neves et al., 2020). Besides, demyelination has been found in the frontal cortex in Alzheimer’s disease patients (Ferrer and Andrés-Benito, 2020), which leaded to cognitive impairment in population at high risk for dementia in Alzheimer’s disease (Kövari et al., 2007; Vanzulli et al., 2020). MBP, as the major component of myelin sheath, its expression level could reflect the level of demyelination. Previous studies have indicated that demyelination, mainly manifested by reduced expression of MBP, was found in aged mice with impaired memory and cognitive ability (Bao et al., 2021). In addition, sevoflurane anesthesia during pregnancy caused the expression of MBP decreased and demyelination in mice, leading to cognitive impairment in the offspring (Zuo et al., 2020). The behavioral function of aged mice was improved when it comes to remyelination, which showed through the up-regulated MBP (Bao et al., 2021). Recent studies have shown that enhanced remyelination reverses cognitive dysfunction in a murine model of Alzheimer’s disease (Chen et al., 2021a). This finding was also confirmed in the chronic cerebral hypoperfusion rat model (Li et al., 2020). In this study, the down-regulation of MBP caused by anesthetic and surgical factors in the hippocampus of aged mice suggesting the appearance of demyelination. Clemastine facilitated remyelination in aged mice with PND, indicating it might be an emerging myelin repair agent, which was consistent with the results of previous studies (Wang et al., 2020a; Xie et al., 2020).

Synapses are places for neuronal function connections occurrence and information transmission. The most common is that the axon terminal of one neuron is connected with the dendrites, dendritic spines or cell bodies of another neuron to form axon-dendritic synapses, axon-spindle synapses or axon-body synapses. Myelin sheath is closely related to axons, and the development of axons is pivotal during the formation of synapse (Han et al., 2017). The synapse is composed of presynaptic membrane, synaptic cleft and postsynaptic membrane. SNAP25 and PSD95 are representative synaptic related proteins, which expressed in the presynaptic and postsynaptic membranes, respectively. Previous studies have shown that the expression level of SNAP25 in exosomes and cerebrospinal fluid of Alzheimer’s disease patients were significantly down-regulated compared with normal counterparts (Jia et al., 2021). The expression level of SNAP25 in the hippocampus of rats with vascular dementia is closely related to the severity of disease (Ren et al., 2018). Furthermore, the significant down-regulated expression level of PSD95 in hippocampus of PND aged rats suggesting that impaired synaptic structure and/or function might have a key role in this persistent defect (Muscat et al., 2021). BDNF is the most abundant neurotrophic factor in the body, mainly expressed in the cortex and hippocampus. Its expression can promote the survival of neurons, increase synaptic plasticity and neurogenesis (Wang et al., 2020b). Previous studies have shown that the expression level of BDNF was significantly down-regulated in hippocampus of mice with PND, which was associated with the impairment of synapse development (Luo et al., 2020). The up-regulation of BDNF was helpful to prevent the occurrence of PND in aged mice (Chen et al., 2018). The expression of PSD95, BDNF and SNAP25 are associated with synaptic plasticity. Previous studies demonstrated that anesthesia and surgery inhibit synaptic function, PND is closely related to synaptic plasticity impairment (Gao et al., 2021). Rescuing the expression of plasticity-related proteins contributes to improve hippocampal-dependent memory deficits caused by anesthesia and surgery (Xiao et al., 2018). In the present study, we further detected the expression of synaptic plasticity-related proteins. The results showed the expression of PSD95, BDNF and SNAP25 were down-regulated in the hippocampus of aged mice with PND, which were reversed after the treatment with clemastine. These results also demonstrate the role of remyelination in the recovery of neuronal function (Del Giovane and Ragnini-Wilson, 2018). The improvement of synaptic plasticity is one of the cores to ameliorate PND.

As previously mentioned, the expression of BDNF can promote the survival of neurons (Wang et al., 2020b), suggesting that synaptic plasticity is related to the survival of neurons (Labandeira et al., 2021; Limanaqi et al., 2021). Meanwhile, previous studies have shown that anesthesia and surgery caused PND by triggering microglia activation and neuron loss (Zhang et al., 2016). This suggests that neuron loss is also involved in the mechanism of PND. Dentate gyrus is the main region of neurogenesis in the hippocampus. In the present study, we found that surgery and anesthesia decreased the number of mature neurons in the hippocampal dentate gyrus though testing the specific marker of mature neurons NEUN. Clemastine treatment could reverse this phenomenon. It suggested that clemastine prevented the loss of hippocampal mature neurons in aged mice with PND. All the above results indicated that clemastine has a positive therapeutic effect on PND.

However, there were some limitations should be addressed in the present study. First, we treated the mice at a daily dose of 10 mg/kg for 2 weeks without analyzing the effects of other drug doses. Although it has been proved in previous studies that the dose has a good safety and remyelination effects (Li et al., 2015; Chen et al., 2021b; Li et al., 2021). Second, we found that clemastine ameliorated PND in elderly mice through a variety of ways, but the underlying mechanism is still unclear and requires further analysis in subsequent experiments. Finally, we found that clemastine ameliorated PND in aged mice, whether it is effective in human remains unknown, which needs to be further confirmed by follow-up clinical studies.

## Conclusion

This study innovatively proposed the presence of demyelination in the pathological process of PND. We identified the ameliorative effect of clemastine on PND by blocking the overactivation of WNT/β-catenin signaling pathway through anti-neuroinflammation to promote OLs differentiation and remyelination. At the same time, synaptic plasticity and survival of hippocampal mature neurons were also improved. Our results might have practical implications and provide new clues and ideas for the clinical treatment of PND.

## Data Availability

The datasets presented in this study can be found in online repositories. The names of the repository/repositories and accession number(s) can be found below: https://www.ncbi.nlm.nih.gov/geo/.

## Glossary

PND: perioperative neurocognitive disorder
TNF-α: tumor necrosis factor alpha
IL-1β: interleukin-1 beta
OLIG2: oligodendrocyte transcription factor 2
MBP: myelin basic protein
PSD95: post-synaptic density protein 95
DLG4: disks large homolog 4
BDNF: brain-derived neurotrophic factor
SNAP25: synaptosomal-associated protein 25
NEUN: neuronal nuclei
OLs: oligodendrocytes; FDA; Food and Drug Administration
GEO: Gene Expression Omnibus
OCT: optimum cutting temperature
PBS: phosphate buffer solution
NFκB: nuclear factor kappa-B
MAPK: mitogen-activated protein kinase
