# Supplementary material for: Clemastine Ameliorates Perioperative Neurocognitive Disorder in Aged Mice Caused by Anesthesia and Surgery
Source: Front Pharmacol. 2021 Aug 23;12:738590. doi: 10.3389/fphar.2021.738590 (PMC8419266; doi:10.3389/fphar.2021.738590)
Supplement: Supplementary file 1 [file Table1.DOCX]

| **Gene** | **Primer sequences (5′–3′)** |
| --- | --- |
| TNF-α | Forward, GGAACCTGCCGGTTTTTGG |
|  | Reverse, CACAGCGAACACCCACGTA |
| IL-1β | Forward, GCAACTGTTCCTGAACTCAACT |
|  | Reverse, ATCTTTTGGGGTCCGTCAACT |
| WNT10B | Forward, GCGGGTCTCCTGTTCTTGG |
|  | Reverse, CCGGGAAGTTTAAGGCCCAG |
| β-catenin | Forward, ATGGAGCCGGACAGAAAAGC |
|  | Reverse, CTTGCCACTCAGGGAAGGA |
| OLIG2 | Forward, TCCCCAGAACCCGATGATCTT |
|  | Reverse, CGTGGACGAGGACACAGTC |
| MBP | Forward, GACCATCCAAGAAGACCCCAC |
|  | Reverse, GCCATAATGGGTAGTTCTCGTGT |
| PSD95 | Forward, ACCAGAAGAGTATAGCCGATTCG |
|  | Reverse, GGTCTTGTCGTAGTCAAACAGG |
| BDNF | Forward, TCATACTTCGGTTGCATGAAGG |
|  | Reverse, AGACCTCTCGAACCTGCCC |
| SNAP25 | Forward, ATCCGCAGGGTAACAAATGATG |
|  | Reverse, CGGAGGTTTCCGATGATGC |
| GAPDH | Forward, GGGTCCCAGCTTAGGTTCAT |
|  | Reverse, TACGGCCAAATCCGTTCACA |

Table 1

The primer sequences used for qRT-PCR.
